# Brain structural network alterations related to serum cortisol levels in drug-naïve, first-episode major depressive disorder patients: a source-based morphometric study

**DOI:** 10.1038/s41598-020-79220-2

**Published:** 2020-12-16

**Authors:** LeHoa Nguyen, Shingo Kakeda, Keita Watanabe, Asuka Katsuki, Koichiro Sugimoto, Natsuki Igata, Takahiro Shinkai, Osamu Abe, Yukunori Korogi, Atsuko Ikenouchi, Reiji Yoshimura

**Affiliations:** 1 Department of Psychiatry, University of Occupational and Environmental Health, Kitakyushu, Japan; 2 Department of Radiology, Graduate School of Medicine, Hirosaki University, Hirosaki, Japan; 3 Open Innovation Institute, Kyoto University, Kyoto, Japan; 4 Department of Radiology, University of Occupational and Environmental Health, Kitakyushu, Japan; 5 Department of Radiology, Graduate School of Medicine, The University of Tokyo, Tokyo, Japan; 6 VNU University of Medicine and Pharmacy, Vietnam National University, Hanoi, Vietnam

**Keywords:** Psychology, Medical research

## Abstract

Higher cortisol levels due to a hyperactive hypothalamic–pituitary–adrenal axis have been reported in patients with major depressive disorder (MDD). Increased cortisol levels change both the brain morphology and function in MDD patients. The multivariate source-based morphometry (SBM) technique has been applied to investigate neuroanatomical changes in some neuropsychiatric diseases, but not MDD. We aimed to examine the alterations in gray matter (GM) networks and their relationship with serum cortisol levels in first-episode, drug-naïve MDD patients using SBM. Forty-two patients with MDD and 39 controls were recruited via interviews. Morning serum cortisol levels were measured, and high-resolution T1-weighted imaging followed by SBM analysis was performed in all participants. The patients had significantly higher serum cortisol levels than the controls. We found two GM sources, which were significantly different between patients with MDD and controls (prefrontal network, p < .01; insula-temporal network, p < .01). Serum cortisol levels showed a statistically significant negative correlation with the loading coefficients of the prefrontal network (r = − 0.354, p = 0.02). In conclusion, increased serum cortisol levels were associated with reductions in the prefrontal network in the early stage of MDD, and SBM may be a useful approach for analyzing structural MRI data.

## Introduction

Major depressive disorder (MDD) is a common and serious mental disorder that negatively affects activities of daily living and is associated with high societal costs and functional impairment^1^. In addition, patients who are depressed have a greater risk of self-harm and even suicide^2^. Regarding MDD pathophysiology, many possible theories have been investigated, including endocrine dysfunction. Studies have shown that the hypothalamus, thyroid, adrenal glands, ovaries, and testes have been linked to depression. Patients with depression have been found to show abnormal responses to thyroid-stimulating hormone and thyrotropin-releasing hormone (TRH), as well as have elevated TRH concentrations in the cerebrospinal fluid and an increased prevalence of antithyroid antibodies^3^. Depressive symptoms are significantly more frequent or severe in patients with subclinical hypothyroidism than in age- and sex-matched controls^4,5^. Episodes of mood disorders have been found to be more prevalent during key life periods with important sexual hormonal changes, such as puberty, menopause, and the postpartum period^6,7^. Higher oxytocin levels are related to fewer depressive symptoms^8^. In addition, oxytocin and vasopressin are also associated with cortisol levels^9^.

Cortisol is a stress hormone that is regulated by the hypothalamic–pituitary–adrenal (HPA) axis and is essential for adequate responses to stressful situations. Alterations in the HPA axis function have been consistently demonstrated as pathophysiological mechanisms of MDD^10^. Patients with MDD have been reported to have cortisol hypersecretion^11^, reduced glucocorticoid receptor mRNA expression, and decreased glucocorticoid-induced inhibitory feedback to the HPA-axis^12^. Previous studies in rats have shown that glucocorticoids increase excitotoxic injury in brain structures that have high concentrations of glucocorticoid receptors, consequently impairing neuro-plasticity^13,14^. According to a meta-analysis, higher levels of cortisol were associated with smaller hippocampal volumes in late-life depression^15^. A neuroimaging study also demonstrated that a high cortisol level was associated with a reduction in the orbitofrontal cortex thickness^16^ and hippocampal volume abnormality^17^. Therefore, elevated cortisol levels may change the brain morphology in individuals with MDD.

Different techniques have been used to assess brain structure abnormalities in MDD patients. The voxel-based morphometry (VBM) approach is a fully automated univariate approach that segments brain images into voxel-wise measures of gray-matter (GM)^18^ and compares changes in voxels over the brain. However, VBM does not consider the relationships among voxels, and it only detects voxels with a specific predicted effect. Diffusion tensor imaging (DTI) is also a useful magnetic resonance imaging (MRI) technique for quantifying and describing microstructural changes in the white matter. Previously, DTI analysis demonstrated that high cortisol levels in MDD were associated with various white matter injuries, including disruptions of the inferior fronto-occipital fasciculus, uncinate fasciculus, and anterior thalamic radiation^19^. However, these data could not provide insight into the interregional connectivity among brain regions. Recently, the source-based morphometry (SBM) technique was applied to investigate neuroanatomical changes in individuals with different neuropsychiatric disorders, such as those with schizophrenia or autism disorder, as well as criminal offenders and patients with delusional infestation^20–24^; however, to date, SBM has not been used to examine individuals with MDD. SBM is a data-driven multivariate approach that combines information across different voxels and identifies unpredicted patterns^25^. SBM applies independent component analysis (ICA) to a segmented image, arranges voxels into sets that contain similar information^25^, and obtains common morphological features of the GM concentration among individuals at the network level. Thus, this method is suitable for identifying novel networks. To the best of our knowledge, no previous study has evaluated the relationships between brain networks and serum cortisol levels in patients with MDD.

This present study examined the associations between neural networks and serum cortisol levels in MDD patients using SBM. The purpose was to determine whether SBM can identify structural brain abnormalities in patients with MDD and whether serum cortisol levels were associated with these alterations. Since antidepressant administration can change brain morphometry^26^, and serum cortisol levels are unstable throughout the day^27^, we included only first-episode, drug-naïve MDD patients in this study and measured cortisol levels after awakening, which is when these levels reach their peak.

## Results

### Baseline demographic data

Demographic information is shown in Table 1. There were no significant differences in age (p = 0.102) and sex (p = 0.2) between the two groups. The MDD group had significantly higher cortisol levels than the healthy subjects (HS) group (p = 0.03).

**Table 1:** Demographic characteristics and values of serum cortisol of participants.

	Healthy subjects	MDD patients	p-value
	(n = 39)	(n = 42)	
Age, years; mean ± SD	43.3 ± 11.6	48.1 ± 14.3	0.1
Male/female	26/13	21/21	0.2
Cortisol level, mean ± SD (nmol/L)	9.6 ± 3.6	12.3 ± 5.1	0.03*

SD, standard deviation, MDD, major depression disorders.

*Significant difference.

### VBM analyses

The whole-brain analysis showed no significant differences in the regional GM volume between the patients and HS.

### SBM analyses

**Figure 1:**
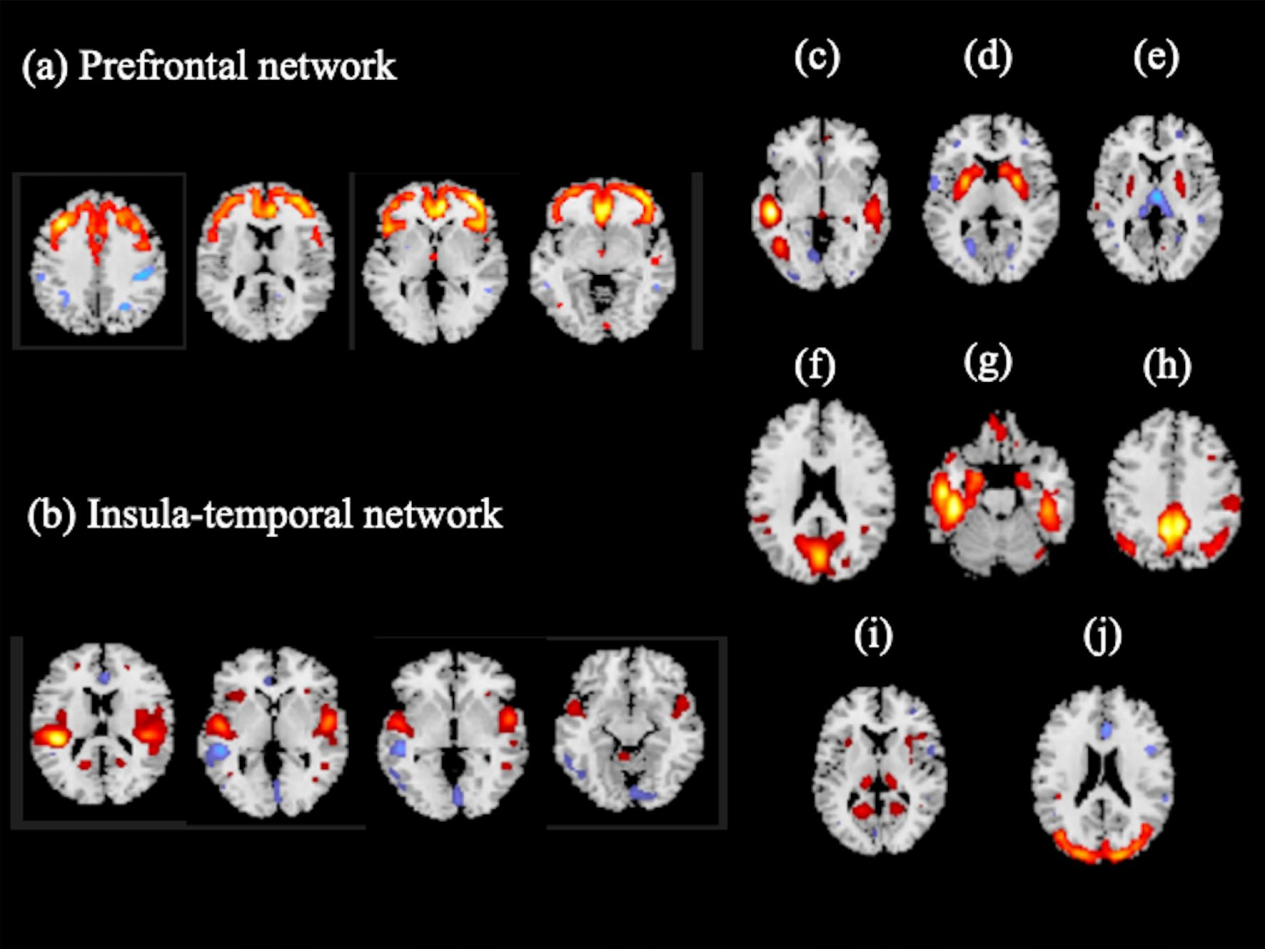
Sources discovered by SBM. Source-based morphometry revealed 10 structural networks. The loading coefficients of the MDD patients in (**a**) the prefrontal network and (**b**) the insula temporal network were significantly lower than those of the healthy subjects (prefrontal network: − 0.25 ± 0.71 and 0.24 ± 0.76, respectively, *p* < 0.01; insula-temporal network: − 0.27 ± 0.80 and 0.39 ± 0.74, respectively, *p* < 0.01). (**c**) component 2; (**d**) component 3; (**e**) Component 6; (**f**) Component 9; (**g**) Component 10; (**h**) Component 11; (**i**) Component 12; (**j**) component 14.

We extracted 17 independent components (ICs). Three of these components were determined to be artifacts based on the criteria that the components contained several sharp edges near the boundary of the brain or primarily appeared in regions that did not contain GM^20^. These components were excluded from subsequent analyses. Thus, from the remaining 14 ICs, we extracted 10 ICs based on a previous review article regarding depression and anxiety^28^. Four ICs, which were excluded from subsequent analysis, mainly included cerebellar networks. Of the 10 ICs, 2 components were significantly different between the MDD and HS group. For these 2 components, the mean loading coefficients were significantly lower in the MDD group than in the HS group after Bonferroni correction (Table 2). We called these components the "prefrontal network" (PF network) (Fig. 1a) and “insula-temporal network” (Fig. 1b).

**Table 2:** Differences in the loading coefficient of independent components.

Components	Healthy subjects	MDD patients	p-value	Cohen’s d
	Mean ± SD	Mean ± SD		
Component 2	0.43 ± 0.54	0.08 ± 0.92	0.04	0.46
Component 3	− 0.28 ± 0.74	− 0.58 ± 0.81	0.09	0.38
Component 4 prefrontal network	0.24 ± 0.76	− 0.25 ± 0.71	< 0.01*	0.68
Component 5 insula-temporal network	0.39 ± 0.74	− 0.27 ± 0.80	< 0.01*	0.84
Component 6	− 0.19 ± 0.74	0.16 ± 0.74	0.04	0.47
Component 9	− 0.20 ± 0.87	− 0.05 ± 0.93	0.46	0.17
Component 10	− 0.05 ± 0.84	− 0.3 ± 0.95	0.21	0.28
Component 11	0.11 ± 0.80	− 0.18 ± 0.72	0.09	0.38
Component 12	− 0.04 ± 0.78	− 0.26 ± 0.85	0.24	0.26
Component 14	− 0.23 ± 0.99	− 0.41 ± 0.86	0.39	0.19

SD, standard deviation, MDD, Major depression disorders.

*Significant difference after Bonferroni correction.

**Figure 2:**
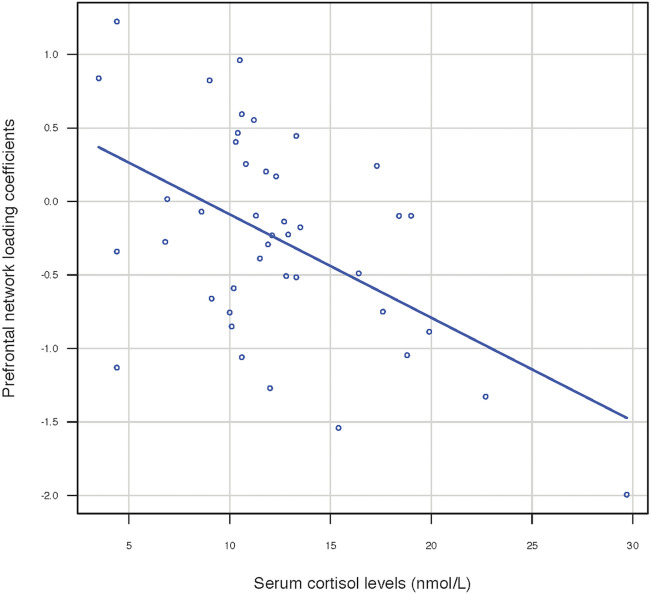
Correlation of cortisol levels and loading coefficients (Z-scores) of the prefrontal network in MDD patients. Scatter plots of prefrontal network loading coefficients and serum cortisol levels in patients with MDD showed a significant negative linear correlation (r = -0.354, *p* = 0.02).

To identify whether serum cortisol levels affected these 2 brain structural networks, we correlated the loading coefficients with the serum cortisol levels for each group. In the MDD group, the serum cortisol levels showed a statistically significant negative correlation with the loading coefficients of the PF network after Bonferroni correction (r = − 0.354, p = 0.0216) (Fig. 2), but the correlation with the insula-temporal network was not significant (r = − 0.294, p = 0.0588). There was no significant correlation in the HS group.

## Discussion

This study revealed two main findings: (1) The loading coefficients of the PF network and insula-temporal network in patients with MDD were significantly lower than those in HS. (2) In the MDD group, only the loading coefficients of the PF network had a significant negative correlation with serum cortisol levels. This is the first study to use a whole-brain morphometric method using ICA, which showed alterations in the GM volume and a correlation between those alterations and serum cortisol levels in first-episode, drug-naïve MDD patients. Thus, our study suggests that the reductions in the GM volume in the PF network in the early stage of MDD were associated with serum cortisol levels and that prefrontal cortex (PFC) alterations might be considered a potential cause rather than a consequence of MDD.

Additionally, we found significantly higher serum cortisol levels in the MDD group than in the HS group. Higher cortisol levels are considered an important characteristic of the pathophysiology of depression. A previous study showed that increased serum cortisol in patients with depression could accurately distinguish between patients with depression and those without depression^29^. Depressive states as side effects of increased glucocorticoid levels have been observed in patients with Cushing’s disease^30^ or accompanying hormone therapy^31^. Treatment of rodents with corticosterone resulted in similar depressive-like behavioral modifications^32^. Mifepristone, a glucocorticoid receptor antagonist, might be effective for the treatment of psychotic depression. This result may support hyperactivation of the HPA axis in MDD pathophysiology.

MDD is a debilitating disease that is characterized by at least one discrete depressive episode lasting at least 2 weeks and involves clear-cut changes in mood, interests and pleasure, changes in cognition, and vegetative symptoms. Because it is described as a heterogeneous syndrome, it is inappropriate to delineate the pathophysiology of MDD by abnormalities in the structure and function of a single brain area, but instead by dysregulation of the interaction of multiple brain regions^33^. Meanwhile, SBM has been used to identify source networks of spatially distinct regions with common covariation among subjects. In turn, these source networks provide the localization of differences in GM concentrations along patterns of voxel-covariation. Notably, such anatomical covariance has been shown to reflect functional connectivity^34^. By using SBM in this study, we found 2 source networks of the GM that show aberrant patterns of covariance in MDD, as compared to HS. Hence, SBM is particularly suitable for studying the pathophysiology of MDD.

The PFC was considered a major part of the circuits that Williams et al. identified as neural circuit dysfunctions underlying depression and anxiety, including the default mode network (DMN), positive affect circuit, frontoparietal attention circuit, and cognitive control circuit^28^. The DMN is primarily composed of the medial PFC, posterior cingulate, lateral temporal cortex, hippocampal formation, and angular gyrus^35,36^. The positive affect circuit consists of the striatal nucleus accumbens and ventral tegmental areas and their projections to the orbitofrontal cortex and medial PFC^37^. The frontoparietal attention circuit is defined by nodes in the medial superior PFC, anterior insula, anterior inferior parietal lobule, and precuneus^38^. The cognitive control circuit is composed of the dorsolateral PFC, anterior cingulate cortex, dorsal parietal cortex, and precentral gyrus. In MDD participants, hyperactivation of the DMN has been associated with higher levels of maladaptive, depressive rumination^39^. Furthermore, the PFC is considered a key node in the frontal-subcortical circuit and frontal-limbic circuit^40^. These circuits are involved in emotional and cognitive processing and have been proposed as pathogenic factors associated with mood and anxiety disorders^41^. A resting-state functional MRI study found decreased functional connectivity among the bilateral hippocampus, dorsolateral PFC, and ventral PFC in the frontal-limbic circuit in first-episode, drug-naïve patients with MDD^42^. Notably, Matsuo et al. provided imaging evidence of prefrontal hyperactivation during working memory tasks in untreated individuals with MDD^43^. The areas of PFC mentioned above were included in the PF network (Fig. 1a), although some other ICs that were revealed in the present study also encompass a part of the prefrontal components.

In this study, we found that the serum cortisol levels were inversely correlated with the loading coeficients of the PF network. This finding suggested that elevated cortisol levels were associated with reductions in the PF network. Cortisol is known to regulate neuronal apoptosis and neurogenesis^44^, and increased glucocorticoids affect both structural and functional changes in the nervous system^45,46^. This finding might be explained by glucocorticoid–glucocorticoid receptor (GR) interactions and regional specificity of GR distribution. Previous animal studies have demonstrated that the cortices containing high concentrations of GR are vulnerable to the neurotoxic effects of glucocorticoids, consequently impairing neuronal plasticity and neurogenesis^14,47^. In the central nervous system, neurons and glia throughout the brain express GR^47^, but the hippocampus and frontal cortex have higher levels of GR expression^47,48^, and the frontal cortex includes the PF network that was identified as being affected in the patients in this study. In a postmortem study, GR mRNA levels showed abnormalities in layers III–VI of the frontal cortex in subjects with depression^48^, which also supports our results.

A potential limitation of our study is the modest sample size, which can lead to some sampling bias. Moreover, although SBM, a multivariate method, takes into account cross-voxel information and is more robust than univariate voxel-wise approaches, this approach should be used on big data to increase the power^20^. However, we limited our sample to first-episode and unmedicated MDD patients so that we could exclude the effects of antidepressants and benzodiazepines on the patients’ brain morphometry. In the future, analysis on a larger number of patients should be conducted to confirm our results from the present study.

## Conclusion

In the current study, SBM, a multivariate statistical data analysis method, was used to investigate differences in GM networks in first-episode, drug-naïve MDD patients, we found changes in the pattern of GM in the PF network and insular-temporal network. However, only the PF network was associated with serum cortisol levels after awakening. These results support the view that the PFC is one of the target sites for the negative-feedback effects of cortisol on the HPA axis in the early development of MDD. Our study also suggests that SBM is a useful alternative to univariate approaches for analyzing structural MRI data.

## Methods

### Participants

A total of 42 first-episode and drug-naïve patients with MDD (21 males, 21 females; mean age, 48.1 ± 14.3 years) were recruited and assessed using the fully Structured Clinical Interview for Diagnostic and Statistical Manual for Mental Disorders, Fourth Edition, Text revision (DSM-IV-TR). Patients were excluded if they met the following criteria: any past DSM-IV-TR Axis I disorder via interviews with a psychiatrist, a history of other medical illnesses, neurological disorders, or use of drugs that may cause depression.

Thirty-nine healthy subjects (HS; 26 males and 13 females; mean age 43.3 ± 11.6 years) were recruited as the HS group via an interview conducted by a psychiatrist using the SCID-I/NP. These subjects were recruited from nearby communities via advertisement and included not only staff at our institution but also their relatives by blood or marriage and close friends. None of the HS had a history of medical or neuropsychiatric illness or a family history of major psychiatric or neurological illness among their first-degree relatives.

### Serum cortisol assay

Because cortisol levels change throughout the day, the timing of a cortisol test is important. In the morning, 30–60 min after awakening, cortisol levels are at their highest^27^. Furthermore, previous studies have shown that patients with MDD exhibit high morning cortisol levels^49,50^. Therefore, 1 h after participants had awakened (9–10 A.M), blood samples were drawn. All samples were immediately centrifuged and the serum was stored at − 20 °C until further analysis. Cortisol was quantitatively displaced from its binding proteins and measured by a direct radioimmunoassay using a highly specific antibody^51^.

### MRI acquisition

All participants underwent brain MRI. The brain images of the patients with MDD were taken before they had received antidepressant medication or psychotherapy. Therefore, all patients were antidepressant-free at the time of the MRI. The MRI data were obtained on a 3.0 T MR system (Signa EXCITE 3T; GE Healthcare, Waukesha, WI) with an eight-channel brain-phased array coil. The images were acquired by 3D fast spoiled gradient-recalled acquisition (3D-FSPGR). The acquisition parameters were as follows: repetition time/echo time/inversion time, 10/4.1/700 ms; flip angle, 10°; field of view, 24 cm; section thickness, 1.2 mm; and resolution, 0.9 × 0.9 × 1.2 mm. All images were corrected for image distortion due to gradient nonlinearity using “Grad Warp”^52^ and for intensity inhomogeneity with the “N3” function^53^.

A radiologist (S.K., 22 years of experience in neuroradiology) who reviewed the conventional MRI data (including T2-weighted images) reported no gross abnormalities, such as infarcts, hemorrhages, or brain tumors, in any of the study participants.

### Image processing for SBM

SBM is a multivariate technique that takes advantage of independent component analysis (ICA). SBM takes into account information across different voxels and identifies unpredicted, naturally occurring patterns of covariance across brain regions^20^. Preprocessing of the images was identical to the procedures adopted for classical VBM analyses, which were introduced in our previous paper^54^.

A fully automatic technique for the computational analysis of differences in regional brain volume throughout the brain was conducted using the SPM12 software program (Statistical Parametric Mapping 12; Institute of Neurology, London, UK)^55,56^. The 3D-FSPGR images in the native space were spatially normalized, segmented into GM, white matter, and cerebrospinal fluid images, and modulated using the Diffeomorphic Anatomical Registration through Exponential Lie Algebra (DARTEL) toolbox in SPM12^57^. DARTEL was proposed by Ashburner as an alternative method for normalization in the SPM package^55^. To preserve the GM and white matter volumes within each voxel, we modulated the images using the Jacobian determinants derived from the spatial normalization by DARTEL. The resulting modulated GM images were smoothed using an 8-mm full-width at half-maximum Gaussian kernel.

The SBM analysis methods have been described in detail elsewhere^20^. For image processing, GIFT toolbox (http://icatb.sourceforge.net) was used^20^. We estimated the number of ICs by using the minimum description length (MDL) principle. The MDL found 17 reliable ICs. Next, we performed ICA using a neural network algorithm (Infomax) that attempts to minimize the mutual information of the network outputs to identify naturally grouping and maximally independent sources^58^. The ICA was repeated 20 times in ICASSO 84 (http://research.ics.aalto.fi/ica/icasso/), and the resulting components were clustered to ensure the consistency and reliability of the results. Reliability was quantified using a quality index, Iq, which ranges from 0 to 1 and reflects the difference between intra-cluster and extra-cluster similarity^59^. All 10 components extracted from the GM images were found to be associated with an Iq > 0.97, indicating highly stable ICA decomposition. SBM involves converting each GM volume into a vector.

As a result, we obtained a matrix where the 81 rows represented the 81 subjects (39 HS and 42 MDD) and each column indicated a voxel. This matrix was decomposed into two matrices by the ICA. The first matrix was named the "mixing matrix" and was composed of a subject per row and an IC per column. The mixing matrix involved "loading coefficients", which demonstrated how each structural component contributed to the 81 subjects and, thus, contained information about the relationship between each subject and each component. The second matrix is named the source matrix, and it specifies the relation between the ICs and the voxels. For GM volume component visualization, the source matrix was reshaped back to a three-dimensional image, scaled to unit standard deviations (Z maps) and thresholded at *Z* > 2.5.

### Statistical analyses

All statistical analyses were performed using EZR software version 1.35 (Saitama Medical Center, Jichi Medical University, Saitama, Japan)^60^, which is a graphical user interface for R (The R Foundation for Statistical Computing, Vienna, Austria). All the results were corrected for family-wise error (FWE).

We applied a two-tailed t-test to assess the differences in age and the Mann–Whitney U test to assess the differences in serum cortisol levels between HS and patients with MDD. A χ^2^ test was used for sex comparisons.

In the VBM analysis, statistical analyses were performed using SPM8 software. Morphological changes in the GM were assessed using a two-sample t-test across diagnostic groups. Age, sex, and total intracranial volume were included as covariates of no interest in all analyses as confounding variables. The differences in GM volume between the patients with MDD and HS were assessed at the whole-brain level. This analysis yielded statistical parametric maps (SPMs [t]) based on a voxel-level height threshold of p < 0.001. We used cluster-level FWE corrections. The significance level was set at an FWE-corrected p < 0.05.

In the analysis of loading coefficients calculated from the SBM, we performed the following analyses: (a) an intra-network comparison between the MDD and HS groups and (b) a linear correlation between intra-network connectivity and cortisol levels. The loading coefficients were transformed to Z-scores using Fisher’s z-transformation. The Z-scores in SBM allowed the identification of sources that exhibit group differences (patients vs. HS) or particular relationships with other variables of interest (e.g., cortisol levels). To compare the intra-network differences, we compared the loading coefficients in each component using two-tailed t-tests. Spearman’s rank correlation was applied to identify associations between serum cortisol levels and the loading coefficients (Z-scores). All the results were thresholded at p < 0.05 corrected with a Bonferroni correction.

### Ethics statement

This study protocol was approved by the Institutional Review Board at the University of Occupational and Environmental, Japan and was conducted in accordance with the Declaration of Helsinki. All participants provided written informed consent after being given a detailed description of the study.

## Data Availability

The datasets generated during and analysed during the current study are available from the corresponding author on reasonable request.
